# Comparison of Sleep Disturbance, Physical Activity, and Health-Related Quality of Life According to Depressive Symptoms in Patients with Metabolic Syndrome: A Secondary Analysis from the Korea National Health and Nutrition Examination Survey Using a Propensity Score Matching Analysis

**DOI:** 10.3390/healthcare11121802

**Published:** 2023-06-19

**Authors:** Jungmi Yun, Yunji Lee

**Affiliations:** 1 College of Nursing, Pusan National University, Yangsan 50612, Republic of Korea; jmyun@pusan.ac.kr; 2 Research Institute of Nursing Science, Pusan National University, Yangsan 50612, Republic of Korea

**Keywords:** sleep, exercise, quality of life, depressive disorder, metabolic syndrome

## Abstract

Metabolic syndrome has become a global epidemic, and the age of its onset is decreasing. However, its prevalence can be reduced by lifestyle modifications. This study examined the differences in sleep disturbance, physical activity, and health-related quality of life associated with depressive symptoms in patients with metabolic syndrome aged ≥ 40 years. This cross-sectional secondary analysis of data from the 2016 and 2018 Korean National Health and Nutrition Examination Surveys. Of 1404 patients with metabolic syndrome aged ≥ 40 years, depressed and non-depressed patients (103 vs. 103) were matched 1:1 on demographic characteristics using propensity score matching. The outcome variables were then compared between the two groups. We investigated health status, including metabolic syndrome indices, health behaviors, such as sleep disturbances and physical activity, and health-related quality of life. After propensity score matching, health-related quality of life was the only variable that differed significantly between the groups; it was significantly lower in patients with depression (0.77) than in those without depression (0.88) (*p* = 0.001). Our results suggest that depression with metabolic syndrome is likely to cause a decrease in patients’ quality of life; therefore, development of management systems and programs for early intervention to tackle at-risk groups is necessary.

## 1. Introduction

Along with the worldwide increase in obesity, metabolic syndrome (MetS) is becoming more common and reaching a global epidemic level [1]. The prevalence of MetS is estimated to range from 12% to 37% in Asian populations and from 12% to 26% in European populations [1]. MetS comprises at least four cardiometabolic abnormalities: hypertension, high fasting glucose levels, abdominal obesity, and atherogenic dyslipidemia [high triglyceride and low high-density lipoprotein (HDL) cholesterol levels] [2,3]. Behavioral and lifestyle factors, such as low physical activity, poor eating habits, drinking, smoking, and stress, as well as genetic factors are associated with MetS. However, lifestyle changes and behavioral modifications, including diet management, self-monitoring of metabolic factors, proper sleep, and physical activity, can help control risk factors and reduce the prevalence of MetS [4,5]. In Korea, 32.1% of adults aged > 30 years have MetS [6]. Furthermore, the prevalence of type 2 diabetes and hypertension, which are significant components of MetS, has increased dramatically in adults over 40 [7]. Therefore, adults aged ≥ 40 years should be targeted in research into factors related to the development of MetS [8], so that early interventions can be based on the relevant factors.

Depression is associated with various diseases, such as diabetes and cardiovascular diseases (CVDs), and its prevalence is higher in those with medical complications than in the general population [9,10]. Increased levels of inflammatory biomarkers, including interleukin-6 and tumor necrosis factor, elevated blood pressure because of sympathetic nervous system activation, and changes in lipid-related markers, such as triglycerides and HDL cholesterol, increase the risk of CVDs in patients with depression [11,12]. These factors affect the risk of developing MetS, and previous studies have reported an association between MetS and depression [13,14,15]. In a large meta-analysis comprising caucasian participants, individuals with depression at baseline had an odds ratio (OR) of 1.52 for the occurrence of MetS later in life. However, those having MetS at baseline had an OR of 1.49 for the occurrence of depression [13]. Moreover, patients with depression are more likely to engage in unhealthy behaviors, such as lack of physical activity, poor dietary habits, smoking, and alcohol misuse. These behaviors can lead to obesity and insulin resistance, which are key factors influencing MetS, and the stress caused by MetS may worsen depressive symptoms [9]. As MetS and depression have a bidirectional relationship, it is necessary to identify and intervene in the presence or absence of depressive symptoms, in patients with MetS.

Sleep disturbances, including insufficient sleep time and decreased sleep quality, can lead to obesity, diabetes, high blood pressure, arteriosclerosis, and CVDs due to increased body mass index (BMI) [16,17]. Increased BMI was associated with all components of MetS [11]. Waist circumference and increased blood sugar are predictive factors for MetS [18] and for the development of depressive disorder [19]. In addition, a direct relationship between sleep patterns and depression [20,21] and MetS [22,23] has also been reported. Sleep is closely related to physical and mental health, and sleep patterns are included as a depression diagnosis component.

Regular physical activity positively affects biological metabolism and helps improve the overall quality of life [24,25]. High levels of physical activity have been shown to improve the health-related quality of life (HRQoL) in women with depression [26]. However, people with depression tend to have significantly lower physical activity levels and spend more time in sedentary activities, such as watching TV or using computers, than do people without depression [27]. In addition, there are reports that patients with chronic conditions and depression are less motivated to make lifestyle modifications and have lower rates of adherence to physical activity recommendations than patients without depression [28]. Therefore, it is necessary to compare the levels of physical activity and HRQoL between patients with MetS, with and without depression.

Individual relationships between MetS and sleep duration, physical activity, and depression as well as factors affecting HRQoL in patients with MetS have been identified previously. However, few studies have compared lifestyle habits and HRQoL in relation to depression in middle-aged patients with MetS. In a previous study that used national-level health data, the selection of participants could not be controlled [29]. This study aimed to examine health status, health behaviors, and HRQoL among patients with MetS aged ≥ 40 years, and their association with levels of depressive symptoms. Hence, the primary research question of this study was: Do sleep disturbances, physical activity, and HRQoL differ according to the presence or absence of depressive symptoms in patients with MetS aged ≥ 40 years? Herein, we investigated the differences between MetS patients with and without depression in terms of relevant variables, using data obtained from the 7th Korea National Health and Nutrition Examination Survey (KNHANES) [30] and propensity score matching (PSM). We chose PSM to reduce selection bias and to control for confounding variables.

## 2. Materials and Methods

### 2.1. Study Design

This study was a cross-sectional and secondary analysis using the 7th KNHANES data to analyze health-related factors according to depressive symptoms among MetS patients. Specifically, we compared sleep disturbance, physical activity, and HRQoL among patients with MetS aged ≥ 40 years.

### 2.2. Data Source and Study Population

We studied patients with MetS aged ≥ 40 years who participated in the 2016 and 2018 Health and Screening Surveys of the 7th KNHANES. The KNHANES is a nationwide survey that produces national-level statistics on health status, health-related awareness and behaviors, and food and nutrition intake of 10,000 Koreans annually [30]. It represents a probabilistic sampling of the general health examination, nutritional status, and physical activity of the Korean population [31]. We used only medical examinations and health-related behavior surveys, excluding nutrition information, such as nutrient intake and dietary behaviors. A sample of 576 survey districts and 13,248 households residing across the country was represented, using population and housing census data, and a stratified colony sampling method.

**Figure 1:**
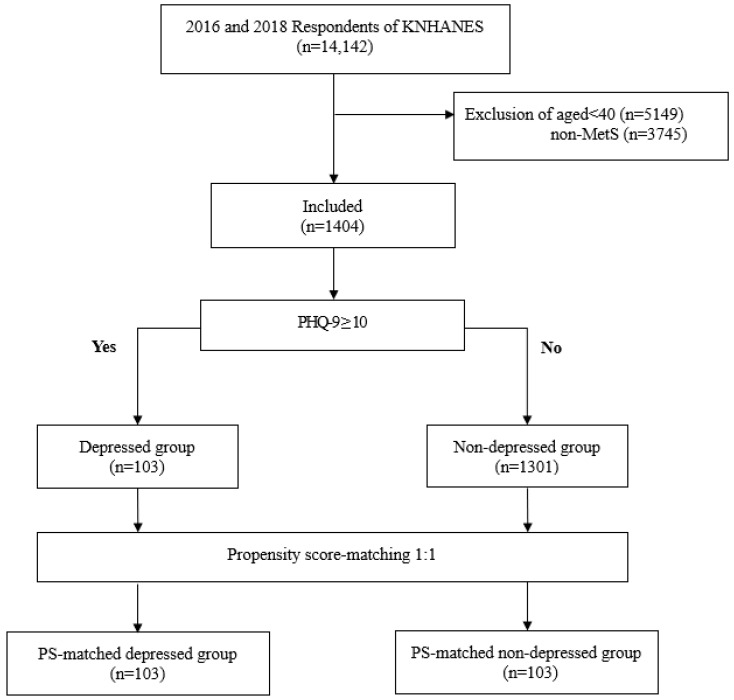
Flow diagram of the study participants. KNHANES, Korea National Health and Nutrition Examination Survey; MetS, metabolic syndrome; PHQ-9, Patient Health Questionnaire-9; PS, propensity score.

A total of 24,269 people participated in the survey: 8150, 8127, and 7992 in 2016, 2017, and 2018, respectively. Depression screening using the Patient Health Questionnaire (PHQ-9) for adults [32] was performed during the first (2016) and third (2018) years of the 7th KNHANES (n = 14,142) [33]. We excluded 5149 individuals aged < 40 years and 3745 individuals who did not meet the diagnostic criteria for MetS, from a total of 14,142 individuals. We classified the depressed group (n = 103; PHQ-9 score ≥10) and non-depressed group (n = 1301; PHQ-9 score < 9) from 1404 individuals who met the inclusion criteria for this study (Figure 1).

### 2.3. Patient and Public Involvement

This study did not involve patients or the public in its design, conduct, reporting, or dissemination plans.

### 2.4. Ethics Statement

We used anonymized data from the 7th KNHANES [30], which was provided by the Korea Centers for Disease Control and Prevention (KCDC) and approved according to the official procedure. This study was exempted from the Institutional Review Board of Pusan National University because there was no direct patient or public involvement, and informed consent was not required.

### 2.5. Outcome Measures

#### 2.5.1. Sociodemographic Characteristics

Eight sociodemographic characteristics: sex, age, household income, residential area, health insurance type, education level, household type, and occupation were extracted from the 7th KNHANES [30]. Household income levels were classified into low, middle-low, middle-high, and high, according to the income quintile group classification of the National Health Insurance premiums [34]. Residential areas were divided into urban and rural, on the basis of administrative characteristics and population size, and occupations were classified as office workers, non-office workers, and unemployed.

#### 2.5.2. Health Status

Health status variables were disease-related characteristics, including systolic blood pressure, diastolic blood pressure, waist circumference, HDL cholesterol, triglycerides, glucose level, BMI, number of comorbid diseases, and subjective health status. Patients with MetS were defined as those with an abnormal status in at least three of the following five indices: hypertension, high fasting glucose levels, abdominal obesity, and atherogenic dyslipidemia (high triglyceride and low HDL cholesterol). Pre-existing, chronic conditions, including hypertension, dyslipidemia, stroke, myocardial infarction or angina, diabetes, renal failure, liver cirrhosis, and osteoporosis were classified as comorbid diseases.

#### 2.5.3. Health Behaviors

Health-related behavior variables included sleep disturbance (average sleep duration per day on weekdays/weekends), physical activity, and sedentary time (usually sitting time per day). Based on metabolic equivalent minutes per week (MET-min/week), the participants were classified into inactive, minimally active, and health-enhancing physical activity (HEPA) groups. The “inactive” group included individuals who performed the least amount of physical activity. The “minimally active” group had those who performed vigorous physical activity for at least 20 min a day for at least 3 days a week, moderate physical activity or walking for at least 30 min a day for at least 5 days a week, or total physical activity of ≥600 MET-min/week. The “HEPA” group included individuals who performed a vigorous physical activity of ≥1500 MET-min/week for at least 3 days per week or those who consumed a total of ≥3000 MET-min/week [35].

#### 2.5.4. Health-Related Quality of Life

The EuroQol (EQ-5D) questionnaire, which provides a simple descriptive profile and a single index value for health status [33], was used to evaluate the participants’ HRQoL. The EQ-5D comprises five dimensions of health: mobility, self-care, usual activities, pain/discomfort, and anxiety/depression [36]. The level of the problem reported in each EQ-5D dimension determines the unique health state. Health states were converted into a weighted health state index by applying scores from the EQ-5D preference weights, drawn from the general population samples [36]. These weights lie on a scale in which total health has a value of 1 and death a value of 0 [36]. In this study, the HRQoL score was calculated by analyzing the five items of the EQ-5D index, according to the weight application criteria of the KCDC.

#### 2.5.5. Depression

The PHQ-9, which consists of 9 questions about the frequency of depressive symptoms over the past 2 weeks, was used to diagnose depression. Each item is rated on a scale from 0 (“not at all”) to 3 (“nearly every day”). The total score range of the PHQ-9 is 0–27, with a higher score indicating higher depression severity. It is classified as mild, moderate, moderately severe, or severe depression by cutoff points of 5, 10, 15, and 20, respectively [32]. In this study, the depressed group was defined as the one PHQ-9 score ≥ 10.

### 2.6. Data Analysis

There was a large difference in the number of samples between the two groups in the classification according to the presence or absence of depression [103 (8.0%) vs. 1301 (92.0%), respectively].

We used PSM to reduce statistical errors. This study was performed in three steps [37]. First, we identified the covariates hypothesized to contribute to the imbalance between the non-depressed and depressed groups. We selected the following covariates for matching: sex, age, education, household income, region of residence, type of health insurance, household type, and employment type. Previous studies have shown that these covariates are demographic factors that influence adult health behaviors according to depression level. A propensity score model was estimated, yielding a propensity score as the probability that each participant was included in the depression group based on the covariates. Respondents in the non-depressed and depressed groups were matched using the same or similar propensity scores. The matching algorithm used the nearest neighbor method with a caliper of 0.05, and the matching ratio was set to 1:1. There are various matching methods for PSMs [34,38,39,40]. We matched the PSM in several ratios and determined that 1:1 matching was the best balance. The standardized mean difference (SMD) values of the matched data were mostly below 0.1, and only education level was below 0.2, which was considered acceptable. After controlling for confounding variables using PSM, we performed a difference test between the variables in the two groups.

Sociodemographic and outcome variables, such as health status (metabolic indicators), health behaviors (including sleep duration on weekdays and weekends, physical activity, and sedentary time), and HRQoL, were summarized using descriptive statistics with frequencies, proportions, means, and standard deviations. Chi-square tests were used for categorical variables and *t*-tests were used for continuous variables to compare differences in sociodemographic and outcome variables between the two groups. The collected data were analyzed using the R program version 4.0.5 and SPSS version 26.0.

## 3. Results

### 3.1. Comparison of Sociodemographics before and after PSM between the Two Groups

The 1414 participants included 565 men (48.5%) and 839 women (51.5%), with an average age of 59.86 years. Regarding the education level, high school graduates accounted for the majority (387 individuals, 31.0%). Regarding health insurance, 1,331 individuals (95.0%) had health insurance and 73 (5.0%) received medical benefits. Regarding family type, 675 individuals (59.4%) lived with their families and 738 (58.8%) were employed (Table 1).

**Figure 2:**
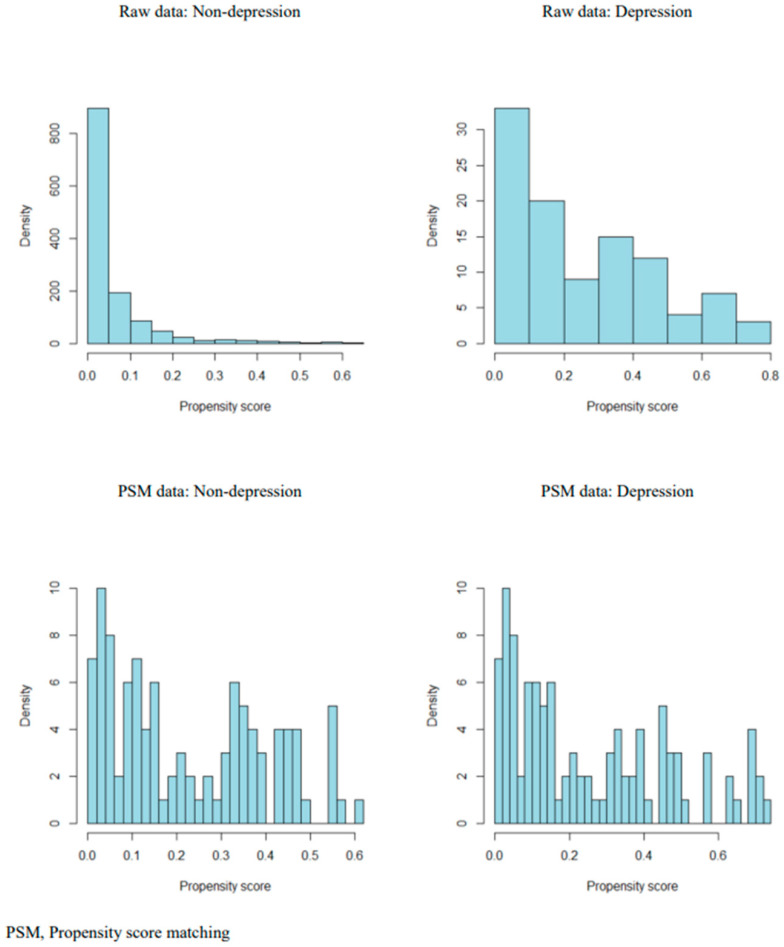
Before and after propensity score matching of two groups.

For PSM between the two groups, a matching balance closer to 0 for a multivariate imbalance is considered well balanced, while a balance closer to 1 is considered a stronger imbalance; thus, the variable cannot be compared [41]. Before matching the depressed and non-depressed groups, there was a significant difference in distribution type. However, a similar distribution pattern appeared after matching, indicating that matching was successful [35] (Figure 2).

Of the 1414 participants in this study, 103 were classified into the depressed group and 1301 into the non-depressed group; a different test was conducted between the two groups. Before PSM, there were significant differences between the general characteristics of the participants, such as household income, health insurance type (*p* < 0.001), and occupation (*p* = 0.002). After PSM, none of the variables showed a significant difference between the two groups (*p* > 0.050). This shows that matching between the two groups was well established (Table 1).

As a result of the subgroup analysis of occupation, there was no significant difference in the average working hours per week and working types (regular and non-regular workers) between the two groups, both before and after PSM (Table 2).

### 3.2. Comparison of Health Status Characteristics before and after PSM between the Two Groups

Among the health status characteristics before PSM, there were significant differences between the two groups in terms of the average number of comorbid diseases and subjective health status. That is, the average number of comorbidities was significantly higher in the depressed group than in the non-depressed group (*p* < 0.001) and as subjective health status decreased, likelihood of depression increased (*p* < 0.001). However, after PSM, there were no significant differences in health status variables between the two groups (Table 3).

### 3.3. Comparison of Health-Behavior Variables and HRQoL before and after PSM between the Two Groups

Before PSM, significant differences were observed in weekend sleep duration and sedentary time across levels of health behavior variables and HRQoL. The rate of depression was higher in participants who slept for an average of 5 or 9 h on weekends than in those who did not (*p* = 0.047). The sedentary time, assessed usually as sitting time all day, was significantly longer in individuals with depression than in those without depression (8.16 vs. 9.47; *p* = 0.002); while the HRQoL was significantly higher in individuals without depression than in those with depression (0.94 vs. 0.77; *p* < 0.001). After PSM, HRQoL was significantly lower in the depressed group (0.77) than in the non-depressed group (0.87; *p* = 0.001). However, no significant differences were found between the two groups in terms of sleep time on weekdays (*p* = 0.488), weekend sleep time (*p* = 0.074), physical activity (*p* = 0.145), or sedentary time (*p* = 0.164). The adjusted *p*-value comparing average sleep duration on weekdays and weekends between the two groups, with age, occupation, working time, and perceived health status as covariates, also showed no significant differences (Table 4).

## 4. Discussion

This study examined the differences in health status, physical activity, sleep disturbance, and HRQoL between MetS patients with and without depression, after adjusting for covariates using PSM. The analysis was based on the data of Korean adults aged ≥ 40 years obtained from the 7th KNHANES, conducted in 2016 and 2018 [30]. The major results of this study are discussed below.

HRQoL was the only variable that showed a significant difference before and after PSM. In this study, HRQoL was lower in the group with depression, which is consistent with the results of previous studies. MetS has been shown to affect the HRQoL. The mean HRQoL of the participants with MetS in the present study was 0.85. This value was lower than that reported in the general population with COVID-19 [42] but higher than that in patients with human immunodeficiency virus [43], skin diseases [44], respiratory diseases [45], and frailty [46]. A cross-sectional study showed that patients with three or more MetS risk factors had higher rates of poor overall health status and physical and mental health than those with one or two risk factors [47]. In another study, individuals with MetS had a higher prevalence of poor health and a higher odds of having more than 14 poor mental health days in the past 30 days compared to those without MetS [48]. A systematic review corroborated these findings and showed that the associations were more pronounced in patients with depression [49]. This suggests that the risk factors for MetS not only directly affect the deterioration of HRQoL, but also affect the burden of comorbid diseases; the stress of managing the illness, and declining health status can affect HRQoL.

A recent study demonstrated that patients with non-alcoholic fatty liver disease experiencing psychological distress were less likely to adhere to a healthy lifestyle [50]. Specifically, they observed a trend of more prominent alterations in psychophysical health status, especially in the affective domains of anxiety, depression, and somatization, in patients who could not adopt lifestyle changes leading to weight loss [50]. This suggests that psychological vulnerabilities, such as depression, are associated with a negative impact on the ability of patients with MetS to make effective lifestyle changes and their HRQoL.

Among the health-related characteristics of participants before PSM, the group with depression had a higher number of comorbidities and a higher rate of perceived poor subjective health status compared to those of the group without depression. However, after PSM, the number of comorbidities was similar, and there were no significant differences in the subjective health status between the two groups. Two studies [9,10] have reported that individuals with other medical conditions have a higher prevalence of depression than that of the general population. This may be due to the number of comorbidities, as well as the burden of the disease owing to its duration and severity. However, caution should be exercised when interpreting the relationship between comorbidities and depression, as this study only examined the number of comorbidities in the patients. In addition, the burden of comorbidities increases with age, and risk of depression increases with increasing burden of comorbidities. It should be noted, however, that patients in the present study were aged ≥ 40 years, which may differ from the age criteria used in previous studies. In previous research, subjective health status was found to be a factor affecting depression severity, and the depressed group perceived their subjective health status more negatively [51]. This study did not consider the severity of depressive symptoms. Therefore, there may not have been a significant difference. However, the subjective evaluation of health status largely influences depression; thus, it should be based on self-active efforts to maintain and manage health status through intensive practical management. For these reasons, it is necessary to provide interventions that simultaneously consider the physical and psychological aspects, to help patients overcome their negative perceptions of chronic diseases, stress, and depression.

Before PSM, there were significant differences in weekend sleep and sedentary times between the groups with and without depression. The rate of depression was high when the weekend sleep duration was <5 h or ≥9 h, and sedentary time was higher in the depressed group. A lack of sleep or excessive sleep is significantly associated with the onset of MetS and depression [18,52]. Results from a study showed that the number of risk factors for MetS increased when the average sleep time per day was <6 h or >8 h [52]. Another study reported that the prevalence of MetS was 1.83 times higher among middle-aged adults with an average sleep time of <6 h than among those with a sleep time of 7–8 h, and prevalence was 1.81 times higher when the average sleep time was ≥8 h [18]. However, as the present study included a secondary analysis of the 7th KNHANES data, various variables related to sleep quality could not be included. Therefore, future studies should evaluate factors related to sleep quality, such as daytime sleep duration and medications. Sleep problems have been reported to exacerbate the emotional control failure in mood disorders, resulting in emotional changes, such as depression, which increases when sleep duration is shorter or longer than the recommended time [51,53]. In a previous study of middle-aged women, depression was the highest among women who slept <5 h, and those with excessive sleep had lower sleep quality because of increased wakefulness and prolonged sleep onset delay [18].

A noteworthy result of our study was that the proportion of patients with depression who sleep for >9 h was higher on weekends than on weekdays (52.0% vs. 58.4%). This is because the participants of this study were middle-aged, socially active individuals, aged ≥ 40 years and with jobs, thus, the lack of sleep during the week was likely compensated for on the weekends. However, whether weekend sleep supplementation reduces the prevalence of MetS remains unclear. A study [20] has shown that adults aged 20–65 years who sleep <6 h a day, on average, have a lower risk of MetS if they receive 1–2 h of sleep on weekends. The group with 1–2 h of sleep supplementation on weekends was more physically active, and had a 45% reduction in the prevalence of MetS. However, in adults aged ≥ 66 years, the risk of MetS increases four-fold when sleeping for >2 h on weekends [23]. In contrast, another study [22] concluded that ad libitum weekend sleep recovery could not be a countermeasure for sleep deprivation and that MetS could not be prevented. In that study, the group with 5 h of sleep per week had reduced insulin sensitivity by 23%, when sleeping too much on weekends. Excessive weekend sleep may be related to emotional changes, such as depression, due to a reduction in physical activity, including leisure and exercise, and a decrease in participation in social activities, indicating that there is a need for strategies for sleep management.

There was no significant difference in physical activity between the two groups in this study; however, it is noteworthy that both groups had high rates of physical inactivity. Considering that the participants of the present study were productive people aged ≥ 40 years, it is likely that employed people spend more time sitting at the office or have less time for regular physical activity. On the other hand, it can be interpreted that the unemployed were less motivated to be physically active even though they had time to exercise. Sedentary lifestyle is a risk factor for MetS. As the amount of physical activity decreases and sedentary time increases, insulin resistance, fasting blood sugar levels, and obesity may increase, causing MetS. Therefore, individualized and tailored interventions are needed to increase the time spent on regular physical activity and to modify unhealthy lifestyle behaviors.

One of the strengths of this study is that it attempted to improve the accuracy of the results by controlling for confounding variables and reducing selection bias using the PSM method with a randomization effect. Based on the KNHANES, which is a large-scale national dataset that is representative of the Korean general population, sleep disturbance, physical activity, and HRQoL outcomes in patients with MetS, with and without depression, were identified. However, this study had some limitations. The variables investigated in this study were based on the results of a self-reported questionnaire. In particular, sleep-related information could not be elucidated further because only weekday and weekend sleep duration were investigated. Therefore, future research should evaluate sleep disorders, including sleep-related quantitative variables, sleep quality, and hygiene. As we only identified differences in health-related variables between the two groups, further research is needed to compare the factors affecting HRQoL in patients with MetS, according to depression status. Despite these limitations, health disparities between the the patients with and without depression have been confirmed in patients with MetS. Since physical and mental health have bidirectional relationships, comprehensive intervention is needed to manage both physical and psychological health, to improve the health and quality of life of patients with MetS.

## 5. Conclusions

In this study of patients with MetS aged ≥ 40 years, depressive symptoms were associated with lower HRQoL, as observed from comparisons between the group with depression and the group without depression. Depression with MetS is likely to cause a decrease in the quality of life of patients; therefore, it is necessary to develop management systems and programs for at-risk patients for early intervention.

## Figures and Tables

**Table 1:** Comparison of sociodemographics before and after propensity score matching between the two groups.

Characteristics	Categories	Total (n = 1404)	Before PSM			After PSM		
			Non- Depressed (n = 1301)	Depressed (n = 103)	*p*	Non- Depressed (n = 103)	Depressed (n = 103)	*p*
		n (Weighted %)/Mean (SE)	n (Weighted %)/Mean (SE)	n (Weighted %)/Mean (SE)		n (Weighted %)/Mean (SE)	n (Weighted %)/Mean (SE)	
Sex	Male	565 (48.5)	530 (48.7)	35 (45.2)	0.561	35 (46.5)	35 (45.2)	0.875
	Female	839 (51.5)	771 (51.3)	68 (54.8)		68 (53.5)	68 (54.8)	
Age (years)		59.86 (0.76)	59.51 (0.41)	60.21 (1.45)	0.650	58.39 (1.51)	60.21 (1.45)	0.395
Education	≤Elementary school	534 (29.4)	482 (28.7)	52 (38.8)	0.312	46 (32.0)	52 (38.8)	0.819
	Middle school	210 (14.5)	194 (14.4)	16 (16.0)		14 (15.8)	16 (16.0)	
	High school	387 (31.0)	365 (31.5)	22 (24.9)		25 (27.0)	22 (24.9)	
	≥College	273 (25.1)	260 (25.4)	13 (20.4)		18 (25.2)	13 (20.4)	
Household income	Low	431 (26.1)	376 (24.4)	55 (48.8)	<0.001	49 (39.0)	55 (48.8)	0.515
	Middle-low	375 (25.7)	344 (25.3)	31 (31.0)		31 (32.4)	31 (31.0)	
	Middle-high	324 (26.2)	313 (27.1)	11 (14.1)		14 (17.1)	11 (14.1)	
	High	274 (22.0)	268 (23.2)	6 (6.0)		9 (11.5)	6 (6.0)	
Residential area	Urban	1063 (81.6)	990 (81.9)	73 (77.9)	0.446	73 (76.7)	73 (77.9)	0.862
	Rural	341 (18.4)	311 (18.1)	30 (22.1)		30 (23.3)	30 (22.1)	
Health insurance type	National health insurance	1331 (95.0)	1247 (96.0)	84 (81.7)	<0.001	88 (89.9)	84 (81.7)	0.088
	Medical care	73 (5.0)	54 (4.0)	19 (18.3)		15 (10.1)	19 (18.3)	
Household type	Alone	729 (40.6)	665 (39.9)	64 (50.1)	0.080	62 (44.8)	64 (50.1)	0.506
	Family	675 (59.4)	636 (60.1)	39 (49.9)		41 (55.2)	39 (49.9)	
Occupation	Office workers	164 (15.4)	159 (15.8)	5 (9.1)	0.002	6 (8.9)	5 (9.1)	0.073
	Non-office workers	574 (43.4)	547 (44.6)	27 (27.7)		41 (46.6)	27 (27.7)	
	Unemployed	666 (41.2)	595 (39.6)	71 (63.2)		56 (44.4)	71 (63.2)	

PSM, propensity score matching; SE, standard of error.

**Table 2:** Subgroup analysis on occupation.

Characteristics	Categories	Total (n = 826)	Before PSM			After PSM		
			Non- Depressed (n = 784)	Depressed (n = 42)	*p*	Non- Depressed (n = 55)	Depressed (n = 42)	*p*
		n (Weighted %) /Mean (SE)	n (Weighted %) /Mean (SE)	n (Weighted %) /Mean (SE)		n (Weighted %) /Mean (SE)	n (Weighted %) /Mean (SE)	
Average working hours per week (h)		39.79 (1.89)	40.14 (0.74)	39.44 (3.69)	0.852	36.95 (2.81)	39.44 (3.57)	0.577
Working types	Regular workers	714 (86.8)	678 (86.9)	36 (84.7)	0.756	49 (90.6)	36 (84.7)	0.451
	Non-regular workers	112 (13.2)	106 (13.1)	6 (15.3)		6 (9.4)	6 (15.3)	

PSM, Propensity score matching; SE, standard of error.

**Table 3:** Comparison of health status characteristics before and after propensity score matching between the two groups.

Characteristics	Categories	Total (n = 1404)	Before PSM			After PSM		
			Non- Depressed (n = 1301)	Depressed (n = 103)	*p*	Non- Depressed (n = 103)	Depressed (n = 103)	*p*
		n (Weighted %) /Mean (SE)	n (Weighted %) /Mean (SE)	n (Weighted %) /Mean (SE)		n (Weighted %) /Mean (SE)	n (Weighted %) /Mean (SE)	
SBP (mmHg)		131.62 (1.26)	131.34 (0.54)	131.90 (2.48)	0.824	133.21 (1.65)	131.90 (2.48)	0.636
DBP (mmHg)		79.79 (0.84)	81.23 (0.36)	78.34 (1.66)	0.091	81.40 (1.42)	78.34 (1.66)	0.188
WC (cm)		90.64 (0.72)	89.94 (0.28)	91.35 (1.38)	0.307	90.82 (1.02)	91.35 (1.38)	0.749
HDL-C (mg/dL) prevalence		41.32 (0.59)	41.98 (0.31)	40.66 (1.14)	0.272	40.89 (1.15)	40.66 (1.14)	0.890
	Yes	527 (36.2)	480 (35.9)	47 (39.5)	0.527	44 (39.6)	47 (39.5)	0.993
	No	877 (63.8)	821 (64.1)	56 (60.5)		59 (60.4)	56 (60.5)	
TG (mg/dL) prevalence		247.74 (13.33)	233.17 (6.02)	262.31 (25.84)	0.271	281.26 (34.03)	262.31 (25.84)	0.655
	Yes	598 (46.9)	551 (46.7)	47 (40.0)	0.589	44 (49.4)	47 (50.0)	0.942
	No	806 (53.1)	750 (53.3)	56 (50.0)		59 (50.6)	56 (50.0)	
Glucose (mg/dL)		120.07 (1.76)	117.10 (1.03)	123.03 (3.34)	0.089	122.64 (5.17)	123.03 (3.34)	0.948
BMI		26.30 (0.11)	26.28 (0.11)	26.58 (0.52)	0.565	26.74 (0.42)	26.58 (0.52)	0.808
Number of comorbid diseases		1.87 (0.10)	1.36 (0.04)	2.37 (0.20)	<0.001	2.08 (0.16)	2.37 (0.20)	0.247
Perceived health status	Very good	53 (4.0)	53 (4.3)	0 (0.0)	<0.001	-	-	
	Good	212 (15.5)	207 (16.3)	5 (5.3)		2 (1.3)	5 (5.3)	0.182
	Moderate	735 (54.0)	715 (56.8)	20 (16.7)		26 (28.2)	20 (16.7)	
	Bad	305 (20.6)	269 (19.2)	36 (39.0)		33 (35.7)	36 (39.0)	
	Very bad	99 (5.8)	57 (3.3)	42 (39.1)		42 (34.8)	42 (39.1)	

BMI, body mass index; PSM, propensity score matching; SBP, systolic blood pressure; DBP, diastolic blood pressure; WC, waist circumference; HDL-C, high-density lipoprotein cholesterol; TG, triglyceride.

**Table 4:** Comparison of health behaviors and health-related quality of life before and after propensity score matching between the two groups.

Characteristics	Categories	Total (n = 1404)	Before PSM			After PSM		
			Non- Depressed (n = 1301)	Depressed (n = 103)	*p*	Non- Depressed (n = 103)	Depressed (n = 103)	*p*
		n (Weighted %) /Mean (SE)	n (Weighted %) /Mean (SE)	n (Weighted %) /Mean (SE)		n (Weighted %) /Mean (SE)	n (Weighted %) /Mean (SE)	
Average sleep duration on weekdays (h)	<5	65 (5.1)	54 (4.7)	11 (10.8)	0.214	6 (5.9)	11 (10.8)	0.488
	5–6	159 (11.7)	144 (11.6)	15 (13.0)	0.741 *	11 (6.5)	15 (13.0)	0.934 *
	6–7	313 (24.7)	295 (25.0)	18 (21.3)		21 (28.6)	18 (21.3)	
	7–8	392 (28.2)	372 (28.5)	20 (23.5)		22 (25.4)	20 (23.5)	
	8–9	301 (20.7)	281 (20.8)	20 (18.2)		25 (21.3)	20 (18.2)	
	≥9	174 (9.7)	155 (9.4)	19 (13.2)		18 (12.3)	19 (13.2)	
Average sleep duration on weekends (h)	<5	50 (3.2)	41 (2.8)	9 (8.7)	0.047	5 (4.4)	9 (8.7)	0.074
	5–6	124 (9.1)	109 (8.9)	15 (11.9)	0.953 *	6 (3.1)	15 (11.9)	0.838 *
	6–7	238 (18.7)	222 (18.8)	16 (17.6)		21 (24.2)	16 (17.6)	
	7–8	374 (26.2)	356 (26.8)	18 (18.6)		26 (31.3)	18 (18.6)	
	8–9	347 (23.8)	329 (24.1)	18 (19.3)		24 (19.8)	18 (19.3)	
	≥9	271 (19.0)	244 (18.6)	27 (23.9)		21 (17.3)	27 (23.9)	
Physical activity	Inactive	1250 (87.8)	1154 (87.4)	96 (91.9)	0.458	91 (84.8)	96 (91.9)	0.145
	Minimally active	135 (10.6)	129 (10.9)	6 (6.0)		12 (15.2)	6 (6.0)	
	HEPA	19 (1.7)	18 (1.7)	1 (2.0)		0 (0.0)	1 (2.0)	
Sedentary time (h)		8.82 (0.22)	8.16 (0.14)	9.47 (0.41)	0.002	8.60 (0.56)	9.47 (0.41)	0.164
HRQoL		0.85 (0.01)	0.94 (0.00)	0.77 (0.03)	<0.001	0.87 (0.02)	0.77 (0.03)	0.001

PSM, propensity score matching; HEPA, health-enhancing physical activity; HRQoL, health-related quality of life; SE, standard error. * Adjusted *p*-value. Covariates: age, occupation, working time (in hours), and perceived health status.

## Data Availability

Data are available in a public, open access repository. The data used in this study are available from the official KNHANES website (https://knhanes.kdca.go.kr/knhanes/sub03/sub03_02_05.do) (accessed on 1 July 2020). The English versions of the data are available online (https://knhanes.kdca.go.kr/knhanes/eng/index.do) (accessed on 1 July 2020).
